# Supplementary material for: Holographic transcranial ultrasound neuromodulation enhances stimulation efficacy by cooperatively recruiting distributed brain circuits
Source: Nat Biomed Eng. 2025 Jul 7;10(1):6–15. doi: 10.1038/s41551-025-01449-x (PMC12647959; doi:10.1038/s41551-025-01449-x)
Supplement: Supplementary file 1 — Supplementary Tables 1–3, Figs. 1–8, methods and references. [file 41551_2025_1449_MOESM1_ESM.pdf]

nature biomedical engineering

Supplementary information https://doi.org/10.1038/s41551-025-01449-x

Holographic transcranial ultrasound neuromodulation enhances stimulation efficacy by cooperatively recruiting distributed brain circuits

In the format provided by the authors and unedited

This file contains:

Supplementary Methods

Supplementary Table 1

Supplementary Table 2

Supplementary Table 3

Supplementary Figure 1

Supplementary Figure 2

Supplementary Figure 3

Supplementary Figure 4

Supplementary Figure 5

Supplementary Figure 6

Supplementary Figure 7

Supplementary Figure 8

Supplementary References

Supplementary Methods: Holographic focused ultrasound

Due to the spherical geometry of our ultrasound array, a multifocal pressure field distribution can be easily achieved splitting the 512 elements of the array into subsets which delays are adjusted to target the desired position. In doing that, the number of elements used to generate a focus change from 512 for single focus to 512/3 ~ 170 for a triangle and 512/5 ~ 102 for a pentagon. As a side effect, one could think the focusing capabilities of the 512/5 subset should substantially vary. We calculated the difference by focusing at a single point shifted -0.5 mm in the x dimension using our calibrated model and different multifocal arrangements (Supplementary Figs. 1–2).

Supplementary Methods: Ultrasound excitation model

1. Pressure field generation

We simulate the field generated by the ultrasound transducer, which was calibrated against transcranial hydrophone measurements, using Field II [1] software in the Rayleigh-Sommerfeld radiation formulation. As described in more detail elsewhere [2], the array's field is estimated adding the contributions of the 512 individual transducers with realistic size and electromechanical impulse response in time domain. To mimic the real array functionality, the delays of the simulated signals are shifted to produce the different multifocal patterns. A volume of 3 x 3 x 3 mm^3 encompasses the pressure distribution at the array's focus with a grid size of 50 µm and 0.011 µs. To calculate time averaged magnitudes, we select two periods at the middle of the 15 cycles of the 3 MHz wave. Thus, the outcome of the simulation is the pressure $p(x, y, z, t)$ dependent of the space and time.

2. Velocity, radiation force, and intensity calculation

Due to the large angular coverage of our array and the complex patterns it can generate, we opted for a general method of calculation and refrained from using simple plane wave approximations to calculate the radiation force and the acoustic intensity. To calculate the radiation force $F = (F_x, F_y, F_z)$ and intensity I, we first need to calculate the particle velocity v. We take the Euler equation [3]

$$\rho \frac{d\mathrm{v}}{dt} = \rho \left[\frac{\partial \mathrm{v}}{\partial t} + (\mathrm{v} \cdot \nabla)\mathrm{v} \right] = -\nabla p, \tag{1}$$

and simplify it for an inviscid fluid, in accordance to the pressure calculation, to obtain

$$v = \int \frac{-\nabla p}{\rho} dt, \tag{2}$$

which has been numerically solved by Fourier series method, based on two periods with 60 samples in total. It follows that the time averaged intensity is obtained from

$$I(x,y,z) = \frac{1}{T_0}\int_0^{T_0} p(x,y,z,t)v(x,y,z,t)dt. \tag{3}$$

Following [4], we calculate the radiation force (force density in N/m³) for an inviscid fluid to second approximation as

$$F_x(x,y,z) = -\rho_0\left[\frac{\partial\langle v_y v_x\rangle}{\partial y} + \frac{\partial\langle v_z v_x\rangle}{\partial z}\right] - \frac{\partial}{\partial x}\left[\rho_0\frac{\langle v_x^2 - v_y^2 - v_z^2\rangle}{2} + \frac{\langle p^2\rangle}{2\rho_0 c_0^2}\right], \tag{4}$$

$$F_y(x,y,z) = -\rho_0\left[\frac{\partial\langle v_z v_y\rangle}{\partial z} + \frac{\partial\langle v_x v_y\rangle}{\partial x}\right] - \frac{\partial}{\partial y}\left[\rho_0\frac{\langle v_y^2 - v_z^2 - v_x^2\rangle}{2} + \frac{\langle p^2\rangle}{2\rho_0 c_0^2}\right], \tag{5}$$

$$F_z(x,y,z) = -\rho_0\left[\frac{\partial\langle v_x v_z\rangle}{\partial x} + \frac{\partial\langle v_y v_z\rangle}{\partial y}\right] - \frac{\partial}{\partial z}\left[\rho_0\frac{\langle v_z^2 - v_x^2 - v_y^2\rangle}{2} + \frac{\langle p^2\rangle}{2\rho_0 c_0^2}\right], \tag{6}$$

where $\langle f\rangle$ denotes the temporal average of f.

3. Activation probability

Directly translating a single number of a given acoustic magnitude to neuronal activation probability becomes difficult when dealing with a complex ultrasound wave field. Thus, we opted for defining a probability density function Ψ that depends on an acoustic magnitude and has to be integrated to yield the activation probability Π.

For simplicity, the probability density is chosen to be separable so that

$$\Psi(p,F,I) = \Psi_p\Psi_F\Psi_I. \tag{7}$$

As our field mostly changes in the two dimensions perpendicular to the arrays' radiation axis, we define the activation probability as

$$\Pi = L\left(\int_S \Psi(p,F,I)dS\right), \tag{8}$$

where L is the sigmoid function

$$L(r) = \frac{1}{1+e^{-\lambda(r-r_0)}}, \tag{9}$$

with λ and r_0 being constant.

3.1 Simulation data input

The time averaged peak pressure $p'(x,y)$ was calculated as the maximum amplitude projection (MAP) of $p(x,y,z,t)$ for $-0.2mm < z < 0.2mm$ and t.

The force density $F'(x,y) = \int_{z_1}^{z_2}|F(x,y,z)|\,dz$ is integrated over same range as peak pressure calculation.

The intensity $I(x,y) \equiv |I(x,y,z_{max})|$ were z_{max} is the plane at which the maximum intensity is reached.

3.2 Thermal effects

Thermal effects are related to the intensity [5] and are included through Ψ_I and were regarded as inhibitory [6], [7].

In case of power heat settings,

$$\Psi_I = 1 - k\left(\frac{\Delta T}{\Delta T_0}\right)^n = 1 - k\left(\frac{I(x,y)}{I_{max}}\right)^n, \qquad (10)$$

where I_{max} denotes the maximum intensity in the space, n is an integer, and k is a constant. In particular, if $n = 1$, it becomes linear

$$\Psi_I = 1 - k\frac{\Delta T}{\Delta T_0} = 1 - k\frac{I(x,y)}{I_{max}}. \qquad (11)$$

Following the experimental results reported in [6] by Darrow et al.,

$$\Psi_I = 1 - \frac{1}{1 + k_1\left(k_2\frac{I(x,y)}{I_{max}} - k_3\right)^n}, \qquad (12)$$

where k_1, k_2, k_3 and n are constants.

In case of ignoring the thermal effects, the temperature-dependent activation probability density

$$\Psi_I = 1. \qquad (13)$$

3.3 Force effects

Assuming a linear dependence between activation probability density and the radiation force yields

$$\Psi_F = \frac{F'(x,y)}{F_{max}}, \qquad (14)$$

where F_{max} denotes the maximum radiation force in space.

In case of logistic dependence of the activation

$$\Psi_F = L\left(\frac{F'(x,y)}{F_{max}}\right). \qquad (15)$$

In case of force model fitting, the pressure-dependent activation probability density is set to

$$\Psi_p = 1. \qquad (16)$$

3.4 Pressure effects

Assuming a linear dependence between activation probability density and the pressure yields

$$\Psi_p = \frac{p'(x,y)}{p_{max}}, \tag{17}$$

where p_{max} denotes the peak pressure in space.

In case of logistic dependence of the activation

$$\Psi_p = L\left(\frac{p'(x,y)}{p_{max}}\right). \tag{18}$$

In case of pressure model fitting, the force-dependent activation probability density is set to

$$\Psi_F = 1. \tag{19}$$

3.5 Model fitting and error

We performed a non-exhaustive model fitting of Π to the experimental data and quantified the errors as mean squared error (MSE) and dynamic time warping (DTW). We explored different configurations for Ψ (Supplementary Fig. 4) and obtained different results for Π (Supplementary Figs. 5–6).

- **Case A: Linear force model without thermal effects**

As shown in Supplementary Fig. 4A, we assumed the force-dependent activation probability density Ψ_F has a linear relationship to the radiation force density and ignored the thermal effects, as shown in Eqs. (13), (14), (16). Following Eqs. (7) – (9), the activation probability yields

$$\Pi = L\left(\int_S \frac{F'(x,y)}{F_{max}} dS\right), \tag{20}$$

where $\lambda = 287$ and $r_0 = 0.0175$ for the sigmoid function L.

- **Case B: Linear force model with power thermal effects**

As shown in Supplementary Fig. 4B, we assumed the force-dependent activation probability density Ψ_F has a linear relationship to the radiation force density and assumed the thermal effects has a power setting, as shown in Eqs. (10), (14), (16). Following Eqs. (7) – (9), the activation probability can be written as

$$\Pi = L\left(\int_S \frac{F'(x,y)}{F_{max}}\left(1 - k\left(\frac{I(x,y)}{I_{max}}\right)^n\right) dS\right), \tag{21}$$

where $k = 0.6$, $n = 3$, $\lambda = 355$ and $r_0 = 0.0171$ for the sigmoid function L.

- **Case C: Logistic force model with Darrow's thermal effects**

As shown in Supplementary Fig. 4C, we assumed the force-dependent activation probability density Ψ_F has a logistic relationship to the radiation force density and assumed the thermal effects has a Darrow's function [5], as shown in Eqs. (12), (15), (16). Following Eqs. (7) – (9), the activation

probability yields

$$\Pi = L\left(\int_S L_F\left(\frac{F'(x,y)}{F_{max}}\right)\left(1 - \frac{1}{1 + k_1\left(k_2\frac{I(x,y)}{I_{max}} - k_3\right)^n}\right)dS\right), \tag{22}$$

where $k_1 = 1$, $k_2 = 6$, $k_3 = 0.2$, $n = -4$, $\lambda = 8$ and $r_0 = 0.5$ for the sigmoid function L_F, $\lambda = 1245$ and $r_0 = 0.0219$ for the sigmoid function L.

- **Case D: Logistic pressure model with linear thermal effects**

As shown in Supplementary Fig. 4D, we assumed the pressure-dependent activation probability density Ψ_p has a logistic relationship to the peak pressure and assumed the thermal effects follow a linear function, as shown in Eqs. (11), (18), (19). Following Eqs. (7) – (9), the activation probability can be written as

$$\Pi = L\left(\int_S L_p\left(\frac{p'(x,y)}{p_{max}}\right)\left(1 - k\frac{I(x,y)}{I_{max}}\right)dS\right), \tag{23}$$

where $k = 0.6$, $\lambda = 13$ and $r_0 = 0.4$ for the sigmoid function L_p, $\lambda = 752$ and $r_0 = 0.0175$ for the sigmoid function L.

- **Case E: Logistic pressure model with power thermal effects**

As shown in Supplementary Fig. 4E, we assumed the pressure-dependent activation probability density Ψ_p has a logistic relationship to the peak pressure and assumed the thermal effects has a power setting, as shown in Eqs. (10), (18), (19). Following Eqs. (7) – (9), the activation probability yields

$$\Pi = L\left(\int_S L_p\left(\frac{p'(x,y)}{p_{max}}\right)\left(1 - k\left(\frac{I(x,y)}{I_{max}}\right)^n\right)dS\right), \tag{24}$$

where $k = 0.4$, $n = 2$, $\lambda = 13$ and $r_0 = 0.4$ for the sigmoid function L_p, $\lambda = 915$ and $r_0 = 0.0183$ for the sigmoid function L.

As a summary, the errors for all cases are listed in Supplementary Table 1. Considering MSE and the insight and simplicity provided by the model, we opted for Case A.

Supplementary Table 1: Summary for USEM model fitting and error

Case No.	Settings			MSE					DTW				
	Ψ_I	Ψ_F	Ψ_p	TUS 1-focus	hTUS 3-foci	hTUS 3L-foci	hTUS 5-foci	Total	TUS 1-focus	hTUS 3-foci	hTUS 3L-foci	hTUS 5-foci	Total
**A**	**Eq. (13)**	**Eq. (14)**	**1**	**0.017**	**0.065**	**0.042**	**0.029**	**0.153**	**0.939**	**0.591**	**0.563**	**0.487**	**2.580**
**B**	**Eq. (10)**	**Eq. (14)**	**1**	**0.014**	**0.063**	**0.043**	**0.044**	**0.164**	**0.945**	**0.542**	**0.590**	**0.514**	**2.591**
**C**	**Eq. (12)**	**Eq. (15)**	**1**	**0.029**	**0.167**	**0.092**	**0.018**	**0.306**	**1.039**	**1.247**	**0.595**	**0.297**	**3.178**
**D**	**Eq. (11)**	**1**	**Eq. (18)**	**0.019**	**0.048**	**0.029**	**0.062**	**0.158**	**0.857**	**0.596**	**0.260**	**0.532**	**2.245**
**E**	Eq. (10)	1	Eq. (18)	0.007	0.077	0.034	0.041	0.159	0.713	0.783	0.349	0.533	2.378

Supplementary Methods: Simplified cortical network model

Supplementary Table 2: Parameters of the simplified cortical network model (sCNM)

Parameter	Symbol	Value	Unit	Source
Membrane capacitance	C_m	1	$\mu F \cdot cm^{-2}$	[8]
Sodium current reversal potential	E_{Na}	50	mV	
Potassium currents reversal potential	E_K	-90		
AMPA current reversal potential	E_{AMPA}	0		
Non-specific leakage current reversal potential	E_{Leak}	-70.3		
Maximal conductance of sodium current at 36°C	$\overline{g_{Na}}$	56	$mS \cdot cm^{-2}$	
Maximal conductance of delayed rectifier potassium current at 36°C	$\overline{g_{Kd}}$	6		
Maximal conductance of slow non-inactivating potassium current at 36°C	$\overline{g_M}$	0.075		
Conductance of non-specific leakage current	g_{Leak}	0.0205		
Adaption time constant of slow non-inactivating potassium current	τ_p	608	ms	
Resting temperature	T_0	36	°C	
Q_{10} temperature coefficient of gating rate constants	$Q_{10,gatin}$	3	-	[9]
Q_{10} temperature coefficient of sodium current maximal conductance	$Q_{10,Na}$	1.4		
Q_{10} temperature coefficient of delayed rectifier potassium current maximal conductance	$Q_{10,Kd}$	4.75		
Rate of thermally activated potassium current conductance increase with temperature	γ_{KT}	4.9	$\mu S \cdot cm^{-2} \cdot {}^{\circ}C^{-1}$	[10]
Pre-synaptic threshold voltage triggering synaptic event	V_{pre}	0	mV	[11]
Synaptic delay	t_{syn}	1	ms	
Rise time constant of AMPA synaptic current	τ_{rise}	0.1		
Decay time constant of AMPA synaptic current	τ_{decay}	3		
Acoustic impedance of brain tissue	Z	1.62	MRayl	[12]
Rate of excitatory drive increase per unit pressure	a	0.56	$\mu A \cdot cm^{-2} \cdot MPa^{-1}$	Educated guess
Steady-state temperature increase per stimulus intensity unit	α_T	0.017	$^{\circ}C \cdot cm^2 \cdot W^{-1}$	
Temperature variation time constant	τ_T	100	ms	
Maximal conductance of AMPA synaptic current (i.e., synaptic weight)	w	90	$mS \cdot cm^{-2}$	
Standard deviation of gaussian noise current	σ_{noise}	2	$\mu A \cdot cm^{-2}$	

Supplementary Table 3: Focus size with respect to the brain volume. Reference number corresponds to the main text reference list.

Species	Ref. #	Brain volume (cm^3)	Focus lateral (cm)	Focus axial (cm)	Focal volume (cm^3)	Volume ratio (%)
**Human**	49	1195	0.45	3.2	0.648	0.05
**Mouse**	This work	0.509	0.05	0.05	0.000125	0.02
**Mouse**	8	0.509	0.16	2.5	0.064	12.57
**Mouse**	9	0.509	N.A.	N.A.	N.A.	N.A.
**Mouse**	10	0.509	0.5 – 0.1	N.A.	N.A.	N.A.
**Mouse**	15	0.509	0.08	0.3	0.00192	0.38
**Mouse**	17	0.509	0.09	0.17	0.001377	0.27
**Mouse**	20	0.509	0.2	0.5	0.02	3.93
**Mouse**	21	0.509	0.44	2	0.3872	76.07
**Mouse**	23	0.509	0.5	N.A.	N.A.	N.A.
**Mouse**	24	0.509	0.3	0.75	0.0675	13.26
**Mouse**	25	0.509	0.57	N.A.	N.A.	N.A.
**Mouse**	26	0.509	0.135	N.A.	N.A.	N.A.
**Mouse**	34	0.509	0.14	0.8	0.01568	3.08
**Mouse**	35	0.509	N.A.	N.A.	N.A.	N.A.

Human brain volume from Cosgrove, K. P., Mazure, C. M. & Staley, J. K. Evolving Knowledge of Sex Differences in Brain Structure, Function, and Chemistry. Biological Psychiatry 62, 847–855 (2007).

Mouse brain volume from Badea, A., Ali-Sharief, A. A. & Johnson, G. A. Morphometric analysis of the C57BL/6J mouse brain. NeuroImage 37, 683–693 (2007).

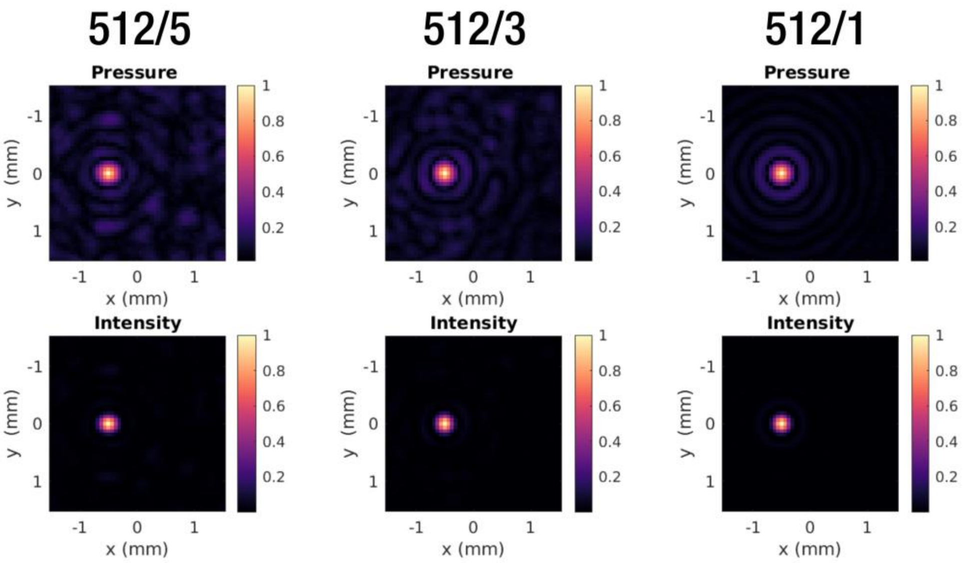

Supplementary Figure 1: Focusing with changing number of array elements. Normalized pressure and intensity distributions at the array's focal plane.

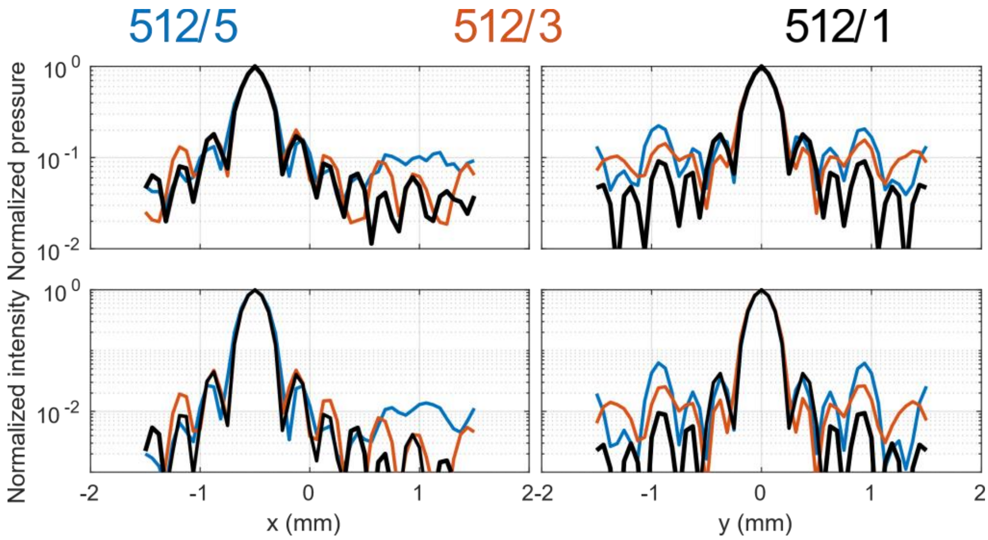

Supplementary Figure 2: Focusing with changing number of array elements: cross sections. Normalized pressure and intensity with a different number of array elements.

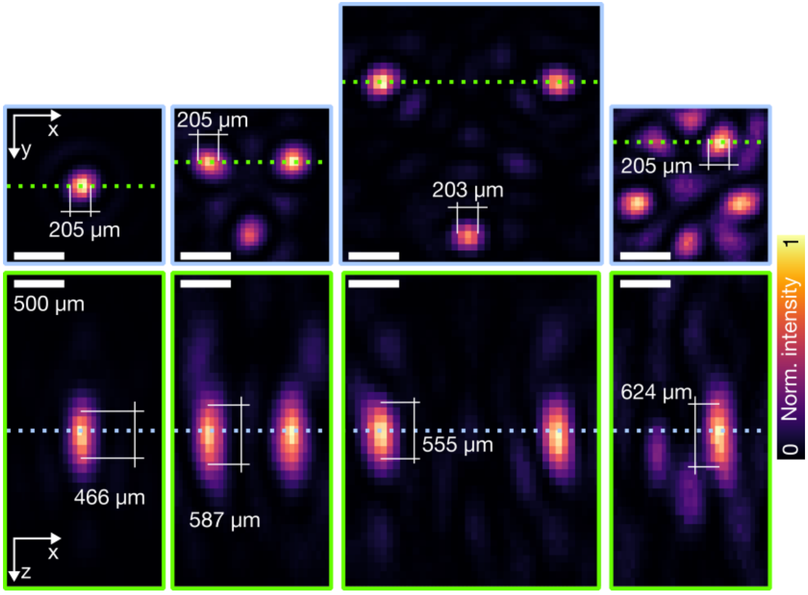

Supplementary Figure 3: TUS and hTUS pressure field measurements. Free-field hydrophone scans for TUS and hTUS pressure fields at 3 MHz. Lateral (top row) and axial (bottom row) FWHM are shown by labels. Scale bars correspond to 500 µm.

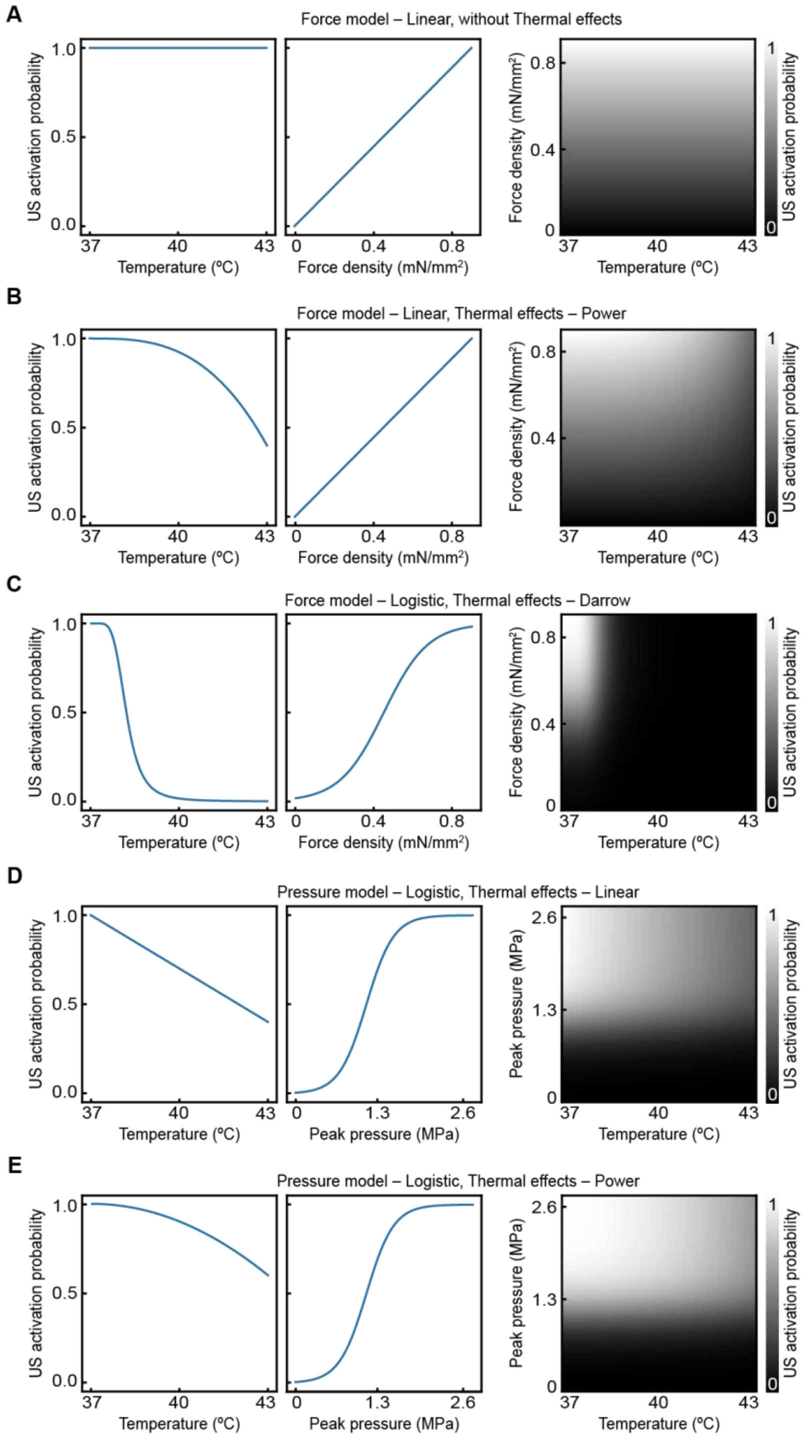

Supplementary Figure 4: Model fitting activation settings. Three cases of force model fitting: (A) linear force model without thermal effects, (B) linear force model with power thermal effects, and (C) logistic force model with Darrow's thermal effects. Two cases of pressure model fitting: (D) logistic pressure model with linear thermal effects and (E) logistic pressure model with power thermal effects. 1D activation settings curves (left two columns) and 2D activation settings map (right column) showing the activation probability density Ψ varying as a function of temperature (intensity) and force density or peak pressure.

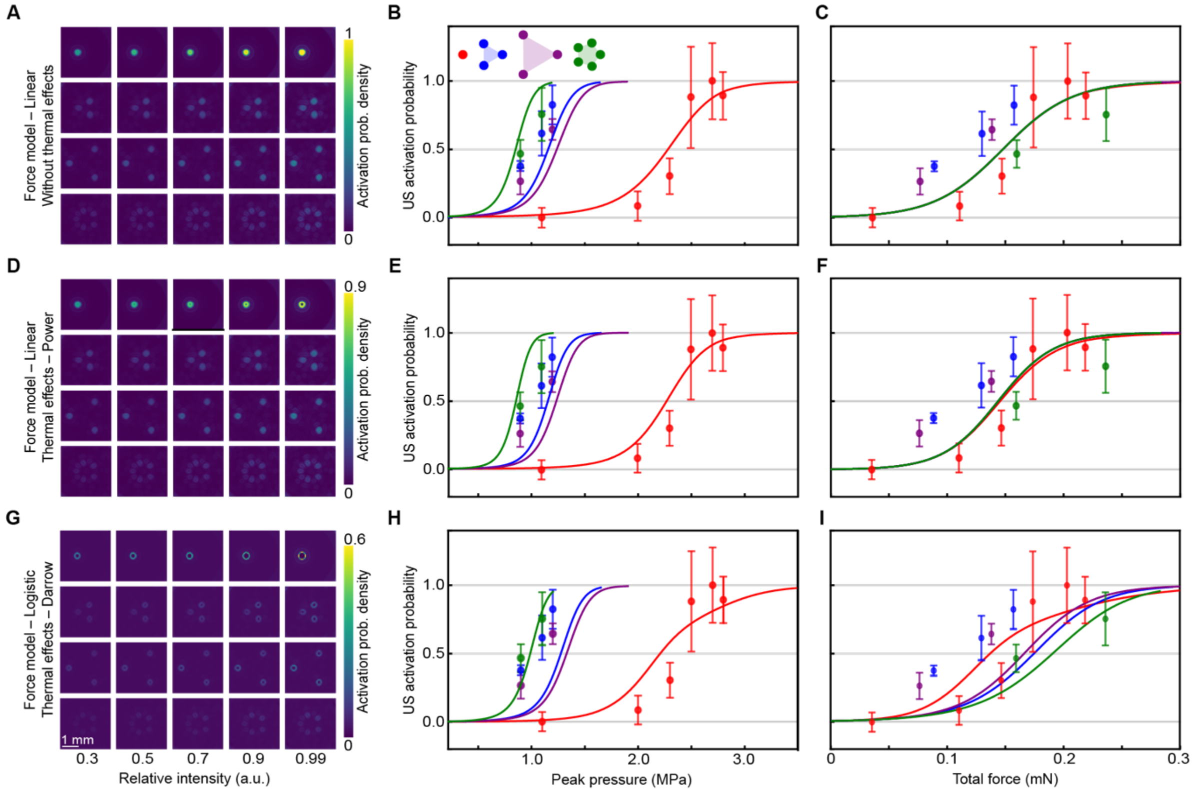

Supplementary Figure 5: Force model fitting results. Three cases of force model fitting: Linear force model without thermal effects (A, B, C), linear force model with power thermal effects (D, E, F), and logistic force model with Darrow's thermal effects (G, H, I). (A, D, G) Activation probability density calculated from the FUS (top row) and hFUS fields for an activation probability indicated by the labels on bottom. Activation probability and experimental data as a function of peak pressure (B, E, H) and total force (C, F, I).

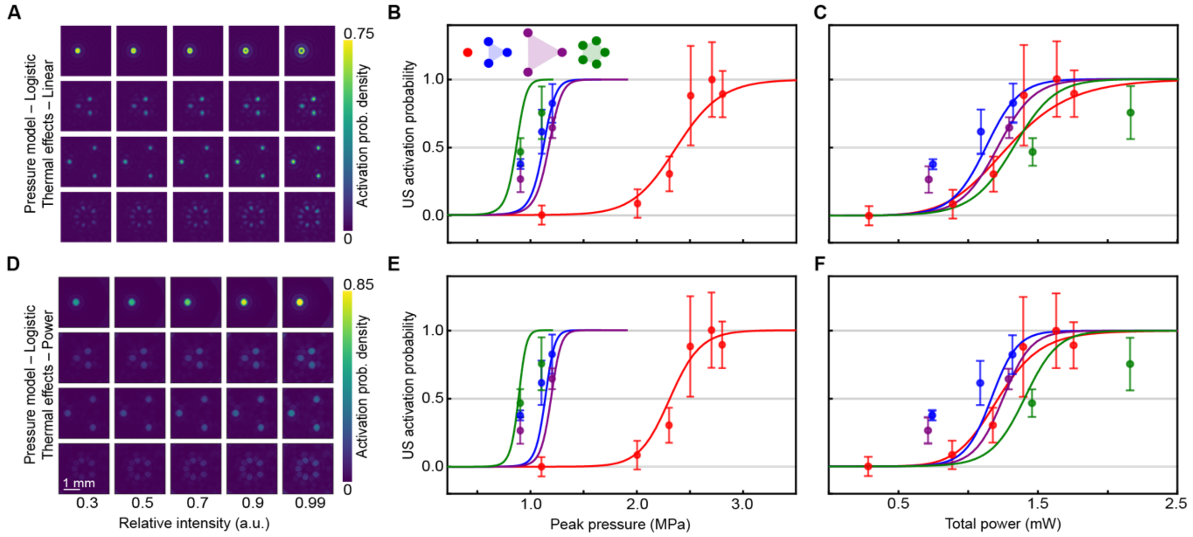

Supplementary Figure 6: Pressure model fitting results. Two cases of pressure model fitting: Logistic pressure model with linear thermal effects (A, B, C) and logistic pressure model with power thermal effects (D, E, F). (A, D) Activation probability density calculated from the FUS (top row) and hFUS fields for an activation probability indicated by the labels on bottom. Activation probability and experimental data as a function of peak pressure (B, E) and total power (C, F).

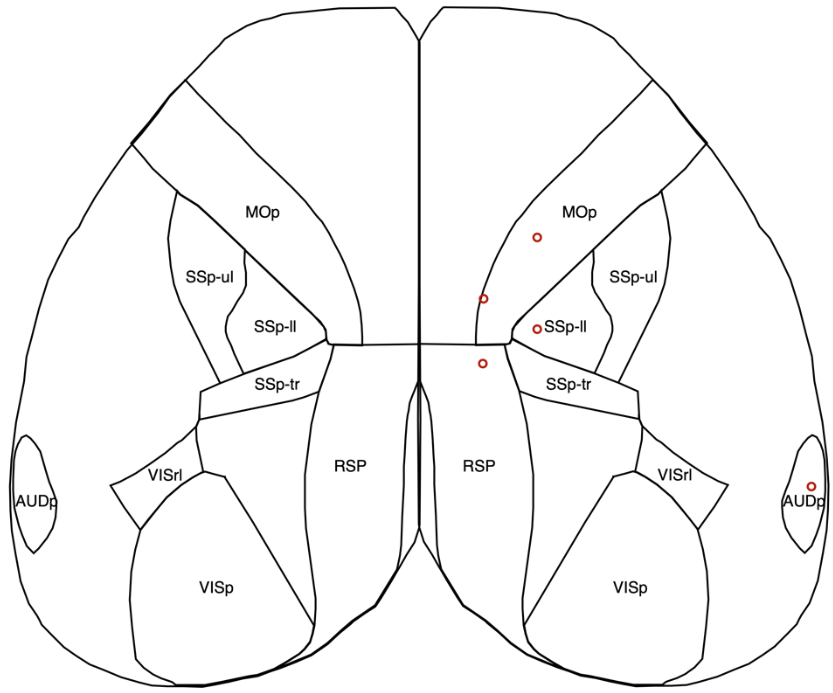

Supplementary Figure 7: Mouse brain schematic showing the regions and ROIs relevant to this study. MOp: Primary motor area, SSp-ul: Primary somatosensory area (upper limb), SSp-ll: Primary somatosensory area (lower limb), SSp-tr: Primary somatosensory area (trunk), RSP: Retrosplenial area, VISrl: Rostrolateral visual area, VISp: Primary visual area, AUDp: Primary auditory area.

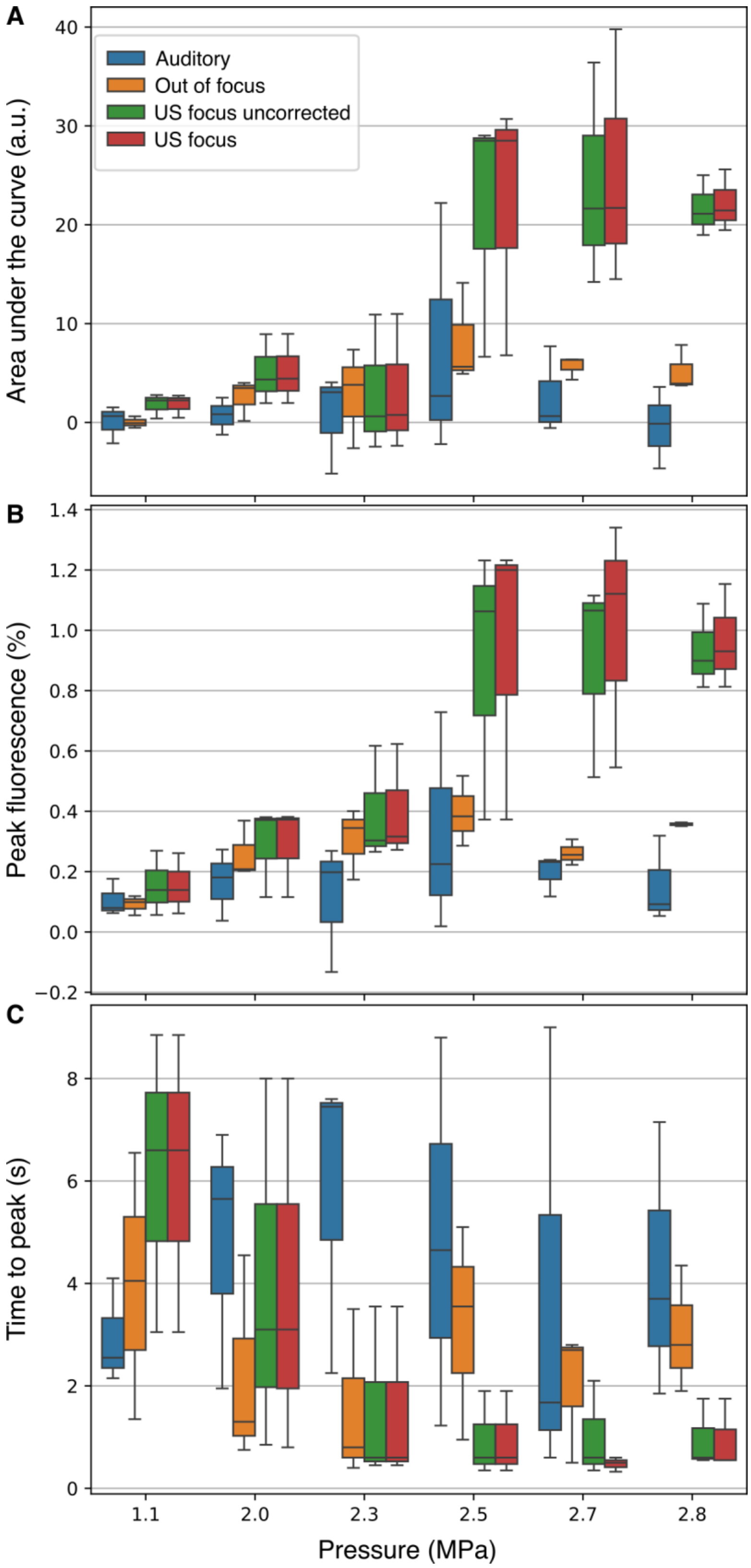

Supplementary Figure 8: Extended single focus activation statistics. A) Change in the area under the time trace at points indicated by the labels as function of the peak pressure between 0.45 and 2 s from the US sonication start. One-way ANOVA analysis (n = 3) performed on three brain regions indicated by labels: Stimulated focus with (F = 5.19, p = 0.009) and without

FTT correction (F=5.51, p = 0.007), out of focus (F=2.36, p = 0.104), and auditory region (F=0.68, p = 0.650). B) Peak fluorescence (between 0.45 and 2 s from the US sonication start) as a function of the peak pressure. One-way ANOVA analysis (n = 3) at the stimulated focus with (F=5.25, p = 0.009) and without FTT correction (F=5.44, p = 0.008), out of focus (F=5.16, p = 0.009), and auditory region (F=0.51, p = 0.758). C) Time to peak as a function of the peak pressure. In all panels, whiskers extend from minimum to maximum, the box from quartiles 25th to 75th, while the horizontal line shows the median.

Supplementary References:

[1] D. Baek, J. A. Jensen, and M. Willatzen, “Modeling transducer impulse responses for predicting calibrated pressure pulses with the ultrasound simulation program Field II,” *J. Acoust. Soc. Am.*, vol. 127, no. 5, pp. 2825–2835, 2010, doi: 10.1121/1.3365317.

[2] H. Estrada, A. Özbek, J. Robin, S. Shoham, and D. Razansky, “Spherical Array System for High-Precision Transcranial Ultrasound Stimulation and Optoacoustic Imaging in Rodents,” *IEEE Trans. Ultrason. Ferroelectr. Freq. Control*, vol. 68, no. 1, pp. 107–115, Jan. 2021, doi: 10.1109/TUFFC.2020.2994877.

[3] L. D. Landau and E. M. Lifshitz, *Fluid Mechanics: Landau and Lifshitz: Course of Theoretical Physics, Volume 6*. Elsevier, 2013.

[4] F. Prieur and O. A. Sapozhnikov, “Modeling of the acoustic radiation force in elastography,” *J. Acoust. Soc. Am.*, vol. 142, no. 2, pp. 947–961, Aug. 2017, doi: 10.1121/1.4998585.

[5] W. D. O’Brien, “Ultrasound–biophysics mechanisms,” *Prog. Biophys. Mol. Biol.*, vol. 93, no. 1, pp. 212–255, Jan. 2007, doi: 10.1016/j.pbiomolbio.2006.07.010.

[6] D. P. Darrow, P. O’Brien, T. J. Richner, T. I. Netoff, and E. S. Ebbini, “Reversible neuroinhibition by focused ultrasound is mediated by a thermal mechanism,” *Brain Stimulat.*, vol. 12, no. 6, pp. 1439–1447, Nov. 2019, doi: 10.1016/j.brs.2019.07.015.

[7] H. Guo *et al.*, “Ultrasound does not activate but can inhibit in vivo mammalian nerves across a wide range of parameters,” *Sci. Rep.*, vol. 12, no. 1, Art. no. 1, Feb. 2022, doi: 10.1038/s41598-022-05226-7.

[8] M. Pospischil *et al.*, “Minimal Hodgkin–Huxley type models for different classes of cortical and thalamic neurons,” *Biol. Cybern.*, vol. 99, no. 4, pp. 427–441, Nov. 2008, doi: 10.1007/s00422-008-0263-8.

[9] M. Ganguly, M. W. Jenkins, E. D. Jansen, and H. J. Chiel, “Thermal block of action potentials is primarily due to voltage-dependent potassium currents: a modeling study,” *J. Neural Eng.*, vol. 16, no. 3, p. 036020, Apr. 2019, doi: 10.1088/1741-2552/ab131b.

[10] S. F. Owen, M. H. Liu, and A. C. Kreitzer, “Thermal constraints on in vivo optogenetic manipulations,” *Nat. Neurosci.*, vol. 22, no. 7, pp. 1061–1065, Jul. 2019, doi: 10.1038/s41593-019-0422-3.

[11] D. Vierling-Claassen, J. A. Cardin, C. I. Moore, and S. R. Jones, “Computational modeling of distinct neocortical oscillations driven by cell-type selective optogenetic drive: separable resonant circuits controlled by low-threshold spiking and fast-spiking interneurons,” *Front. Hum. Neurosci.*, vol. 4, p. 198, 2010, doi: 10.3389/fnhum.2010.00198.

[12] IT’IS Foundation, “IT’IS Database for thermal and electromagnetic parameters of biological tissues.” Feb. 22, 2022. doi: 10.13099/VIP21000-04-1.
